# The effect of metformin in clinical studies on knee osteoarthritis: an updated systematic review and meta-analysis

**DOI:** 10.3389/fmed.2025.1652732

**Published:** 2025-10-16

**Authors:** Xiaoqi Guo, Haibin Xie, Yuanfan Liu, Jianye Hou, Haochen Tan, Lin Miao, Ying Xu, Ye Tian

**Affiliations:** ^1^Department of Orthopedics, Shengjing Hospital of China Medical University, Shenyang, Liaoning, China; ^2^Department of Breast Surgery, Cancer Hospital of Dalian University of Technology, Cancer Hospital of China Medical University, Liaoning Cancer Hospital and Institute, Shenyang, Liaoning, China; ^3^Department of Anesthesiology, Shengjing Hospital of China Medical University, Shenyang, Liaoning, China

**Keywords:** knee osteoarthritis, metformin, total knee arthroplasty, pain management, meta-analysis

## Abstract

**Objectives:**

Metformin is regarded as a potential drug for the treatment of knee osteoarthritis (KOA), but its efficacy remains unclear. This systematic review and meta-analysis aims to evaluate the efficacy of metformin in the treatment of KOA.

**Methods:**

According to Preferred Reporting Items for Systematic Reviews and Meta-Analyses (PRISMA) guidelines, we searched three literature databases, including PubMed, Web of Science and EMBASE, and conducted a systematic review and meta-analysis of randomized controlled trials and cohort studies up to April 1, 2025, studies evaluating the use of metformin in the treatment of KOA were included. Hazard ratio (RR), standard mean difference (SMD), and the corresponding 95% confidence interval (CI) were used as aggregated statistics. This systematic review is registered on RROSPERO (CRD420251026750).

**Results:**

A total of six eligible studies included 4,628 patients (1,489 patients use metformin versus 3,139 patients non-use). The main observation outcome was the score of the pain scale (WOMAC, KOOS, VAS, etc.) of the knee joint and the incidence of total knee arthroplasty (TKA) is a secondary outcome. Analysis of the included population the included population reveals that the BMI levels of all individuals were at the overweight range. In this situation, the comprehensive results show that regardless of different conventional treatment methods, courses of treatment and doses, combined metformin treatment in the incidence of joint replacement [RR = 0.65, 95% CI (0.54, 0.77), *p* < 0.00001] and knee pain scores [SMD = −0.47, 95% CI (−0.84, −0.11), *p* = 0.01], it was superior to the control group treatment.

**Conclusion:**

The research results show that metformin can relieve the pain of KOA with high BMI and reduce the possibility of joint replacement. However, due to the less of randomized controlled trials (RCTS), clinicians should be fully cautious when using it.

**Systematic review registration:**

https://www.crd.york.ac.uk/PROSPERO/view/CRD420251026750.

## 1 Introduction

Knee osteoarthritis (KOA) is a common degenerative joint disease characterized by progressive cartilage degeneration, synovitis and changes in subchondral bone, which leads to knee pain and functional impairment, exerting significant pressure on the quality of life and mental health of patients, and also imposing a heavy economic burden on society (1). In the past few years, the treatment of OA has remained a challenging task for doctors. At present, the treatment methods for OA are limited to pain-relieving drugs, such as acetaminophen and non-steroidal anti-inflammatory drugs. The only available option for patients with advanced OA is joint replacement surgery (2). Given the increasing prevalence of OA, especially among the elderly and individuals with metabolic disorders such as obesity and diabetes, there is an urgent need for effective treatment methods that can not only alleviate symptoms but also change the progression of the disease.

Metformin is one of the most widely used hypoglycemic drugs in the world. As a first-line drug for treating type 2 diabetes, some literature indicates that it has the effects of anti-aging and alleviating aging-related diseases (3). A study has shown that metformin activates adenosine monophosphate-activated protein kinase (AMPK), which plays an important role in mediating its benefits by regulating inflammation and cellular metabolism. This activation leads to a reduction in inflammation, promotes the survival of chondrocytes, and weakens cartilage degeneration (4). Following this, another study also confirmed that metformin can activate the AMPK-autophagy-lysosome pathway to promote inflammatory cell death and thereby protect cartilage. Moreover, in the rat model, metformin can alleviate OA pain by inhibiting pain mediators (such as CGRP) (5).

Therefore, in recent years, the potential application of metformin in the treatment of OA has attracted more attention. In addition to relevant animal experiments, some clinical studies have also shown that metformin is a promising candidate drug for the treatment of OA. A cohort study reported that compared with sulfonylurea drug treatment, the incidence of OA in patients with type 2 diabetes treated with metformin was significantly reduced, and the risk of developing OA was decreased by 24%, demonstrating the effect of preventing OA (6). However, it is not clear to what extent these results can be generalized to a broader OA population, especially non-diabetic patients.

A systematic review published in 2022 expounded on the efficacy of metformin in the treatment of OA in preclinical and human studies. This review did not conduct a meta-analysis and was different from our search strategy (7). Moreover, there were three updated studies after 2022. Therefore, we combined the latest experimental studies and pool-up analyses. Systematically evaluate the efficacy of metformin in patients with KOA.

## 2 Methods

This systematic review and meta-analysis was conducted in accordance with the Preferred Reporting Items for Systematic Reviews and Meta-Analyses (PRISMA) guidelines (8), aiming to determine the efficacy of metformin in patients with KOA. The PRISMA checklist is shown in Supplementary Table S1 of the supplementary document. And we prospectively registered in the International Prospective Register of Systematic Reviews (PROSPERO). The registration number is CRD420251026750.

### 2.1 Search strategy

We conducted a systematic literature search in PubMed, Web of Science and Embase, retrieving the published English articles up to April 1, 2025. In the search method, a combination of MeSH terms and free words were used, and the following terms are employed to retrieve the database: “Metformin,” “Dimethylbiguanidine,” “Dimethylguanylguanidine,” “Glucophage,” “osteoarthritis,” “Osteoarthritides,” “Osteoarthrosis,” “Osteoarthroses.” The detailed search strategy is shown in Supplementary Table S2.

### 2.2 Inclusion and exclusion criteria

The inclusion criteria that should be met are: (1) The study type is a randomized controlled trial, a cohort study, or a case–control trial; (2) The research subjects were adult patients with KOA, regardless of whether they were complicated with metabolic syndrome (such as diabetes, etc.); (3) The research content is the effects of metformin use and non-use on KOA patients; (4) At least one outcome measure should be included (knee Pain Scale, number of joint replacement surgeries, WOMAC Scale, KOOS scale, Knee Function Scale, etc.). Exclusion criteria: (1) Non-human studies, reviews, letters, case reports, laboratory articles, and conference abstracts; (2) No reliable data; (3) Non-English articles. The included studies were independently discovered and evaluated by two researchers. Any differences between the two investigators were resolved by consensus.

### 2.3 Data extraction

Data extraction was independently completed by two researchers and organized in spreadsheets. Any differences that arise shall be reasonably decided by additional researchers. We mainly focus on the following data in the researches: author, publication date, study design, study country, study duration, sample size, participant age, gender, body mass index (BMI), WOMAC score, Knee pain score, and KOOS score. When the continuous variables in the study were using the median range or interquartile range, we calculated the corresponding mean ± standard deviation through the reported mathematical methods (9, 10). If the data in the research is missing or not reported, we contact the relevant authors to obtain the completed data.

### 2.4 Quality assessment

We used the Newcastle—Ottawa Scale (NOS) (11) to assess the quality of cohort studies and case–control studies. The score range was 0–9 points, and a score of 0–3 points indicated a lower quality of the articles, 4–6 indicates average article quality, while a score of 7–9 indicates relatively high quality. For randomized controlled trials, we used the modified Jadad scale (12) to assess the risk of bias. A score of 1–3 was considered low quality, and a score of 4–7 was considered high quality.

### 2.5 Statistical analysis

We used Revman 5.4.1 (Cochrane Collaboration, London, United Kingdom) and Stata 17.0 (StataCorp LP, College Station, TX, United States) to collect and analyze the data. For the continuous variables in the outcomes, we used the standardized mean differences (SMD) to combine the trial results using different scales; For binary categorical variables, risk ratios (RR) was used as the outcome report, and the corresponding 95% confidence intervals (CI) were given for all outcomes, *p* < 0.05 was considered statistically significant. The I^2^ test was used to determine the level of heterogeneity. If I^2^ = 0, it was considered that there was no heterogeneity. When I^2^ > 50%, significant heterogeneity is considered. The results are summarized using the random effects model. When I^2^ < 50%, the fixed effects model is applied. Finally, we conducted a sensitivity analysis to ensure the reliability of the results. For publication bias, we used Stata for Egger’s test, *p* < 0.05 was considered to have significant publication bias.

## 3 Results

### 3.1 Literature search and study characteristics

**Figure 1:**
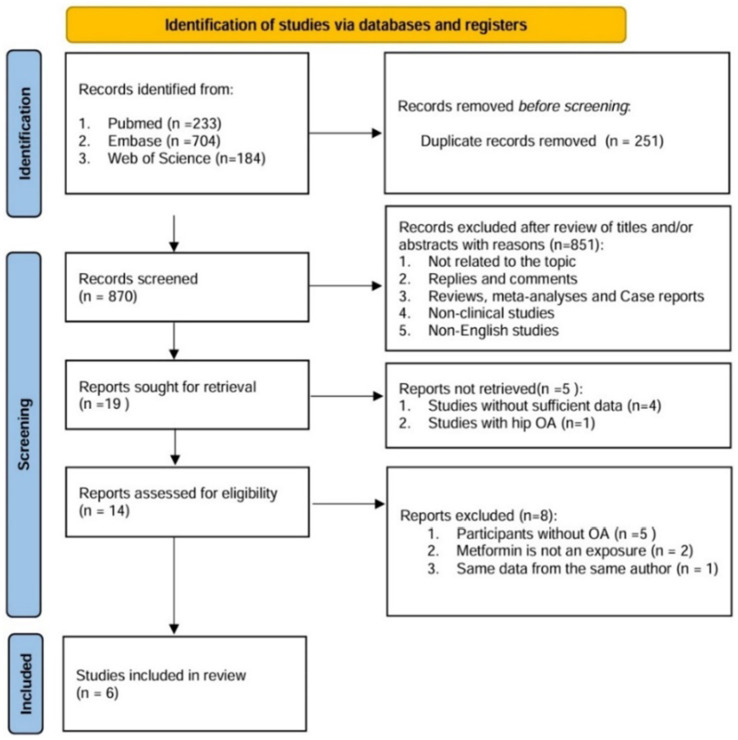
PRISMA flow diagram of study selection process.

Through literature retrieval, we included a total of 1,121 relevant literatures in PubMed (*n* = 233), Web of Science (*n* = 184), and Embase (*n* = 704). The process flow chart of literature retrieval and selection is shown in Figure 1. Literature management was conducted using Endnote X9 software. After deleting 251 duplicate articles, two researchers reviewed the titles and abstracts of the remaining 870 articles. After excluding articles irrelevant to the research topic, non-clinical and non-English ones, there were 19 remaining articles. Finally, a detailed full-text reading was conducted on these 19 articles, and a total of six studies were included in our review. We extracted the relevant characteristic data, including research methods, participant information, intervention measures, outcome indicators, etc. The summary of the relevant materials included in the study is shown in Table 1. A total of 4,628 patients (1,489 with metformin and 3,139 without) were included in the combined analysis. Of these articles, two were prospective cohort studies (13, 14), two were retrospective cohort studies (15, 16), and two were randomized controlled trial studies (17, 18).

**Table 1:** Data collection.

Study	Region	Study design	Mean follow-up	Age (year)		Sex (male)		BMI		Sample size (n)		Pain scores		Joint replacement	
				Metformin	No-metformin	Metformin	No-metformin	Metformin	No-metformin	Metformin	No-metformin	Metformin	No-metformin	Metformin	No-metformin
Aiad et al. (17)	Egypt	RCT	12 weeks	51.00 ± 5.752	52.28 ± 6.100	3	3	36.40 ± 5.555	38.49 ± 5.934	25	25	−4.32 ± 1.8246	−1.72 ± 1.7929	NA	NA
Alimoradi et al. (18)	Iran	RCT	4 months	49.3 ± 9.3	47.2 ± 9.0	10	14	29.1 ± 3.1	29.0 ± 3.8	44	44	−15.9 ± 18.9517	1.3 ± 19	NA	NA
Wang et al. (21)	USA	Prospective	6 years	62.3 ± 7.7	61.7 ± 8.5	15	270	34.9 ± 3.8	33.9 ± 3.1	56	762	−0.9 ± 4.1998	−0.6 ± 3.7997	3	88
Halabitska et al. (14)	Ukraine	Prospective	3 months	48 ± 14.074	47 ± 13.148	19	15	28.02 ± 2.8148	29.87 ± 2.8963	34	26	−1 ± 1.4815	−1 ± 1.9333	NA	NA
Lu et al. (15)	CN Taiwan	Retrospective	10 years	70.17 ± 10.85	70.74 ± 10.56	415	830	NA	NA	968	1936	NA	NA	124	314
Chen et al. (16)	China	Retrospective	19 years	72.7 ± 10.1	72.7 ± 10.7	83	80	25.8 ± 4.3	25.8 ± 4.4	362	346	1.6 ± 1.9	2.1 ± 1.7	20	72

### 3.2 Quality assessment

Two RCTS were evaluated using the Jadad scale, the modified Jadad score range for qualified studies is from 4 to 7, and both scores were within the range, the detailed scoring criteria are presented in Table 2. The remaining four articles were evaluated using the NOS scale. Studies with a score of 7–9 were considered of high quality. Two literatures are regarded as high-quality studies. It is presented in Table 3.

**Table 2:** The modified Jadad’s scores scale for RCTS.

Modified jadad’s scores scale for randomized controlled trials						
Study	Randomization	Concealment	Blinded	With or drop-out		Total
Aiad et al. (17)	2	1		2	1	6
Alimoradi et al. (18)	1	1		2	1	5

**Table 3:** The NOS scale for cohort study.

Author	Study quality of included studies based on the Newcastle-Ottawa scale								Quality score
	Study quality of cohort studies								
	Selection				Comparability		Outcome		
	Representativeness of the exposed cohort	Selection of the non-exposed cohort	Ascertainment of exposure	Demonstration that outcome of interest was not present at start of study	Comparability of cohorts on the basis of the design or analysis	Assessment of outcome	Was follow-up long enough for outcomes to occur	Adequacy of follow up of cohorts	
Wang et al.	*	*	*	*	**	*	*	*	9
Halabitska et al.	*	*	*	*	*	*	*		6
Lu et al.	*	*	*	*	*	*		*	6
Chen et al.	*	*	*	*	*	*	*	*	8

Each * is worth one point. Except for “Comparability”, which can be scored up to 2 points, the remaining items can each be scored up to 1 point. The total score ranges from 0 to 9, with a higher score indicating a higher quality of the research.

### 3.3 Effect on knee pain

**Figure 2:**
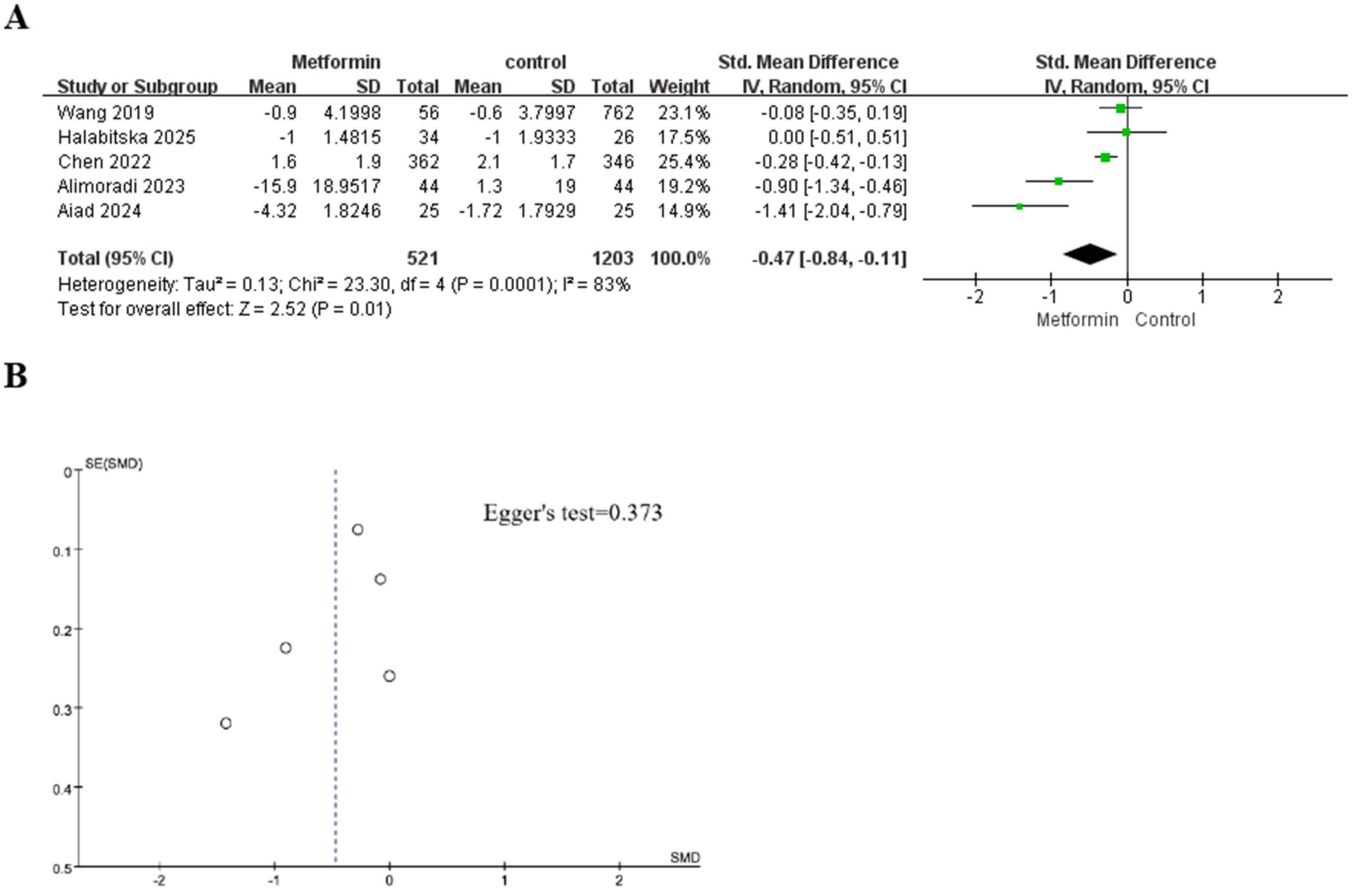
**(A)** The effect of metformin-use on knee pain. **(B)** Funnel plot and Egger’s test for the knee pain.

A total of five studies were included in the analysis, involving 1724 patients (521 cases using metformin and 1,203 cases not). The heterogeneity test indicated significant heterogeneity among the studies (I^2^ = 83%). Therefore, the random effects model was used to calculate the SMD and 95% confidence interval. The aggregated results showed that under conventional treatment, compared with the group without metformin treatment, the knee pain score of patients treated with metformin was significantly reduced. It decreased by 0.84 in the metformin group and by 0.11 in the non-metformin group (SMD: −0.47; 95% CI: −0.84, −0.11; *p* = 0.01). The specific situation is shown in Figure 2A.

Although only five studies were included, we still plotted the funnel plot and conducted Egger’s test. The subjective assessment of the funnel plot revealed a mild publication bias. However, Egger’s test indicated no statistical significance (Figure 2B for details).

### 3.4 Effect on total knee arthroplasty

**Figure 3:**
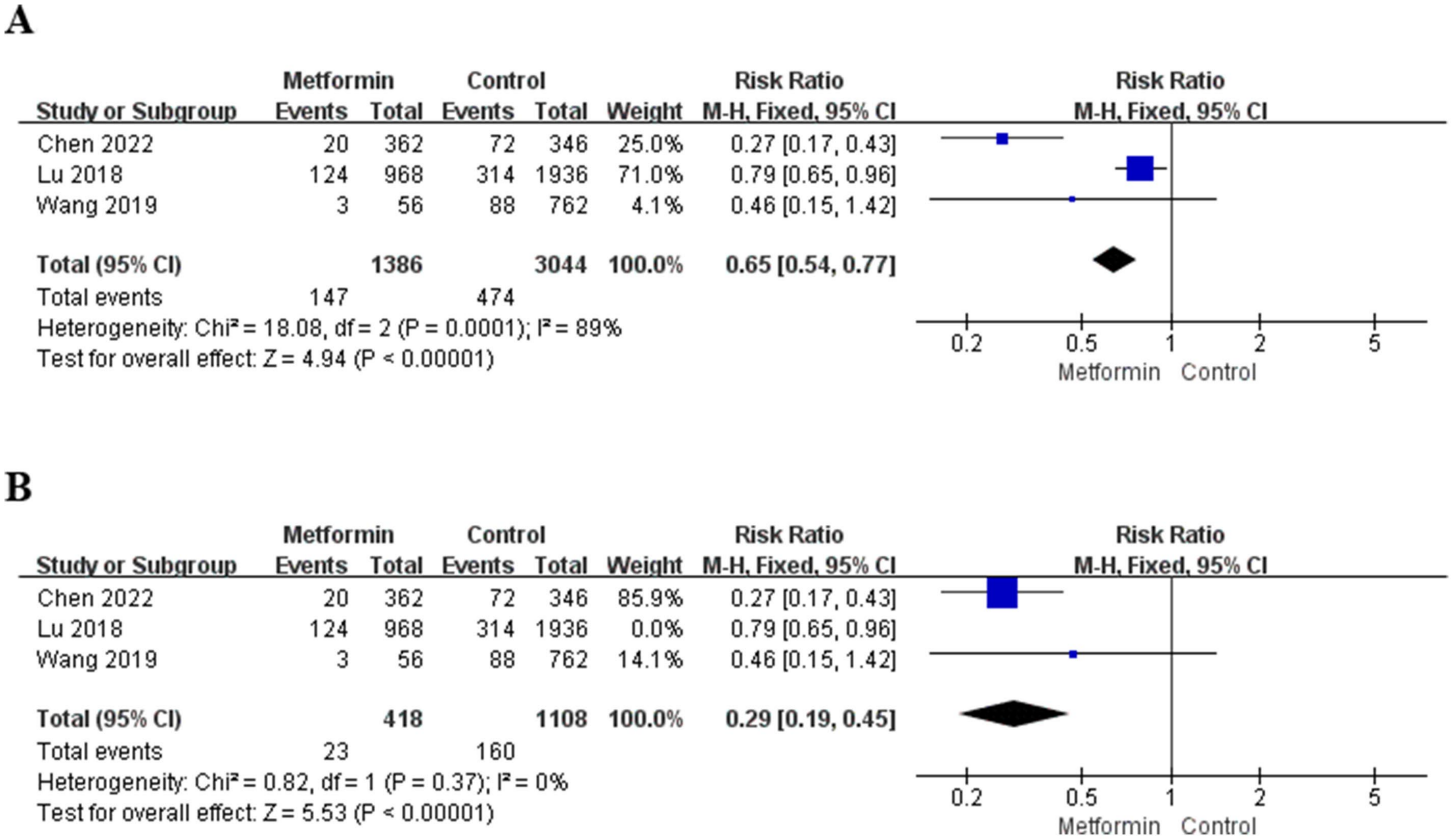
**(A)** Effect on total knee arthroplasty. **(B)** The forest plot after removing one study.

A total of three studies were included in the analysis, involving 4,430 patients (1,386 patients used metformin and 3,044 patients non-use). The risk ratio (RR) and 95% confidence interval were calculated using the fixed-effect model. The aggregated results suggested that the risk of TKA after metformin treatment was significantly reduced (RR = 0.65; 95% CI: 0.54, 0.77; *p* < 0.00001), and the specific situation is shown in Figure 3A. However, the heterogeneity in the study was significant (I^2^ = 89%). Therefore, the three studies were analyzed, respectively. When the study of Lu et al. (15) was not included, the heterogeneity decreased to zero, and the results were also statistically significant, as shown in Figure 3B.

### 3.5 The influence of follow-up time on the knee pain

**Figure 4:**
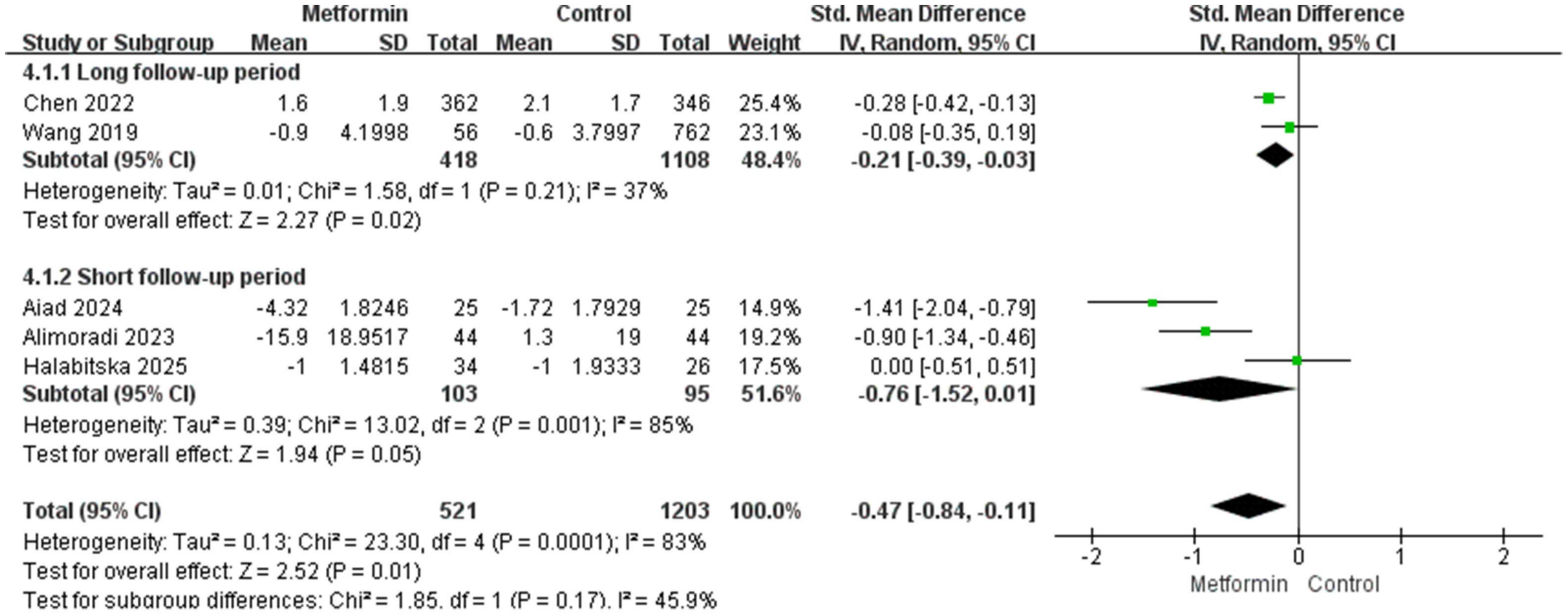
Follow-up time on the knee pain.

We believe that a follow-up period of more than 5 years is long enough. Therefore, we conducted a subgroup analysis to compare the effect of metformin on pain in terms of usage duration. The aggregated results showed that the metformin use group was able to reduce the pain score more during the long follow-up period. The results were statistically significant and the heterogeneity was also lower (SMD: −0.21; 95% CI: −0.39, −0.03; *p* = 0.02, I^2^ = 37%), and in terms of short-term pain management, the results were not statistically significant (SMD: −0.76; 95% CI: −1.52, 0.01; *p* = 0.05, I^2^ = 85%), as shown in Figure 4.

### 3.6 The influence of research type on the results

**Figure 5:**
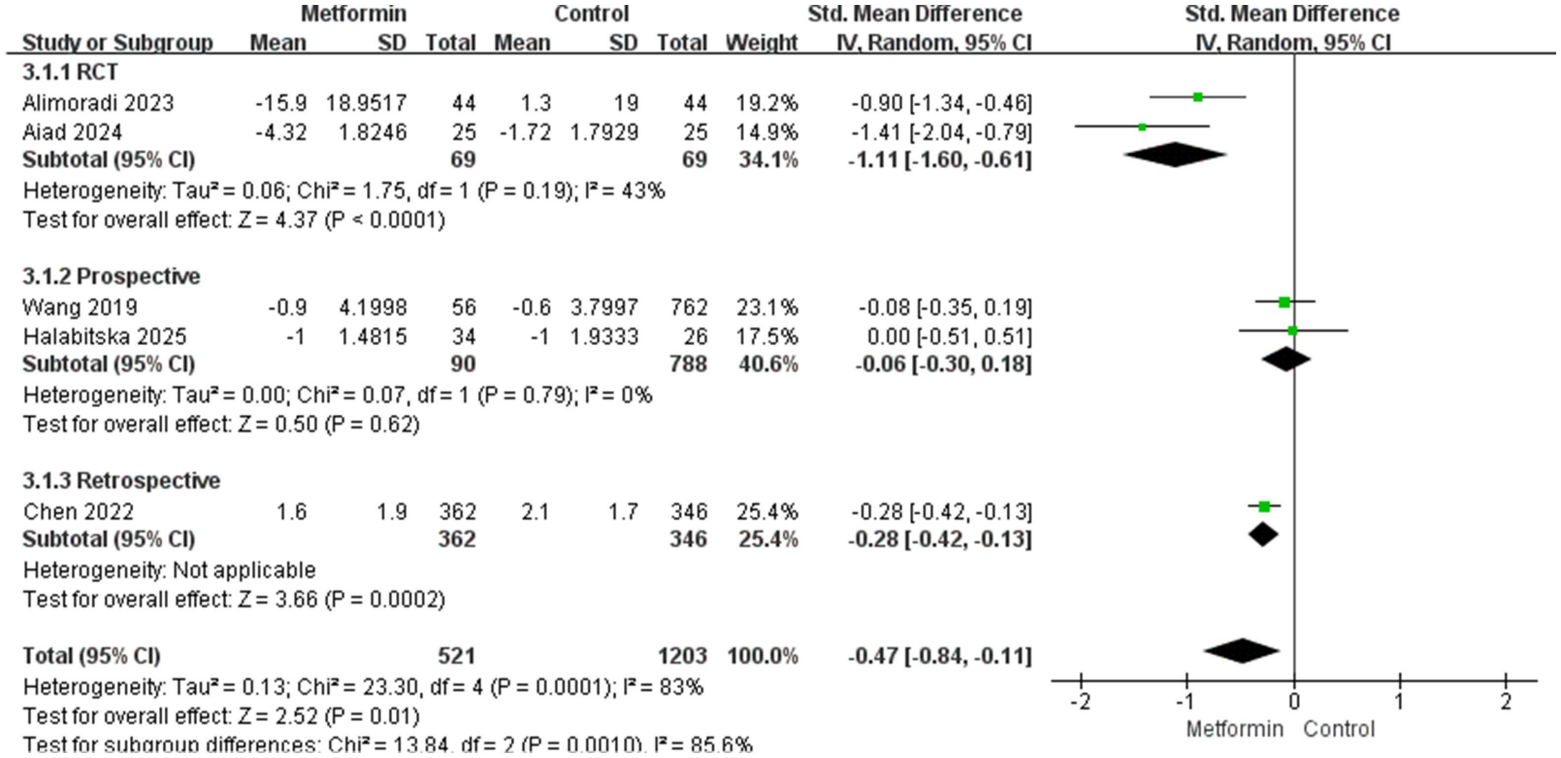
Research type on the results.

Two RCT experiments were analyzed, and the results were statistically significant with moderate heterogeneity (SMD: −1.11; 95% CI: −1.60, −0.61; *p* < 0.0001, I^2^ = 43%). In the two prospective studies, the results did not suggest statistical significance (SMD: −0.06; 95% CI: −0.30, 0.18; *p* = 0.62, I^2^ = 0%), subgroup difference test: χ^2^ = 13.84, df = 2 (*p* = 0.0010), I^2^ = 85.6%, suggesting that the inhibitory effect of metformin on pain varies in different types of studies, which may be related to the experimental design protocol. Moreover, cohort studies are not as strict in controlling variables as RCTS. The details are shown in Figure 5.

## 4 Discussion

Metformin, as one of the most common hypoglycemic drugs. It has been shown to mitigate inflammation by metformin attenuates the migration of M1 macrophages to the chondrocytes *in vitro*, and diminishes the infiltration of M1 macrophages in OA synovial tissues, thereby potentially reducing joint inflammation associated with OA progression (19). Another study shows that metformin can promote autophagy in cells and enhance normal mitochondrial function, eliminate aging-related proteins and damaged organelles, improve the function of T cells, reduce the release of inflammatory factors (such as Th17-related cytokines), and delay age-related inflammation (20). Furthermore, in animal experiments, metformin in the mouse OA model enhances the mitochondrial autophagy mediated by PINK1/Parkin by activating SIRT3. This process clears damaged mitochondria, reduces oxidative stress and inflammation, and thereby protects chondrocytes (21). Therefore, supported by a variety of preclinical studies, in order to determine its clinical efficacy, we performed the latest systematic review and pooled analysis of 6 comparative studies including 4,628 patients, and our results revealed several important findings.

First, due to the different pain score scales in the outcome indicators, we used SMD for analysis and because of the excessive heterogeneity, a random effects model was adopted. The analysis results of knee joint pain showed that the pain score of the metformin use group decreased significantly compared with the control group. However, this might be due to the selection bias caused by the fact that all the people included in the study were overweight (BMI ≥ 25). Although there is no definite conclusion on the effect of metformin on body weight to date, many studies have still found that metformin can moderately reduce body weight, especially among the diabetic population, metformin is believed to have a positive impact on weight loss (22, 23). In addition, Obese people have a higher risk of KOA. Some studies have shown that weight loss may help prevent the occurrence of symptomatic diseases and relieve existing symptoms (24, 25). This can prove that due to the weight loss effect of metformin on overweight people, the incidence of KOA has been reduced. There is currently no data to support whether metformin can play the same role in people with normal weight.

Second, analyses of the follow-up time of the disease course for patients with KOA can prove that the pain-suppressing effect produced by metformin in long-term application is better than that in short-term use. This might be because patients who use it for a long time lose weight more significantly, and due to the patients’ self-management and treatment, the progression of arthritis can be delayed. However, after comprehensive analysis, the heterogeneity of the results is too high. So, we conducted subgroup analyses for different research types, the results suggest that RCTS studies have moderate heterogeneity (I^2^ = 43%), while the heterogeneity of prospective studies is 0. Considering comprehensively, the difference in research types and the length of the follow-up period may be the main source of heterogeneity.

Third, some studies suggest that in the diabetic population, the use of metformin can reduce the risk of developing OA and decrease the possibility of knee replacement (6, 26). Therefore, we considered whether it could also play a role in the general population and conducted a summary analysis. An analysis was conducted on 4,430 patients in three studies that included the outcome measure of TKA. The results suggested that the use of metformin could reduce the risk of joint replacement (RR = 0.65; 95% CI: 0.54, 0.77; *p* < 0.00001; I^2^ = 89%). By conducting a detailed analysis of these three studies one by one, it can be found that when the study of Lu 2018 (15) was excluded, the heterogeneity was significantly reduced (Figure 3B) (I^2^ = 0), this study can explain most of the sources of heterogeneity. An analysis of this article reveals that the included population encompasses not only KOA but also hip arthritis. Therefore, it can also explain why there is high heterogeneity.

Our study provided evidence-based medicine evidence through meta-analysis that compared the knee pain and TKA occurring of metformin use and non-use in patients with OA. However, we must acknowledge several limitations of the present study. Primarily, there were only six studies included in our pooled analysis, and among them, there are only two RCTS. Most studies included in retrospective or prospective cohort items were included without proper confounders control. Furthermore, significant heterogeneity was observed in outcomes. Although we have attempted to explain the source of heterogeneity, we still cannot determine its true source. As a result, considering the potential influence of such uncontrolled confounders, the results should be interpreted with caution. Secondly, although we intended to include the entire population in the analysis, we found that all the study subjects were individuals with high BMI. This limits the generalizability of the research results.

Despite these limitations, our study has several notable advantages. We completed the latest meta-analysis, confirming the efficacy of metformin in KOA, especially significantly reducing knee pain levels in overweight and obese patients with a high BMI. However, due to the lack of high-quality articles included, the application of this meta-analysis has been affected. Therefore, in order to further determine the therapeutic effect of metformin on KOA, more strictly designed, large-scale, long-term follow-up randomized controlled trials are needed.

## 5 Conclusion

Overall, the latest systematic review and meta-analysis indicate that metformin has potential therapeutic effects in KOA, offering benefits in pain relief, improved joint function, and potentially slowing the progression of the disease, especially among those with a high BMI population. However, researchers also pointed out that the current clinical evidence is insufficient, and more high-quality, large-sample, and long-follow-up clinical studies are needed to confirm these findings and better understand the optimal dose and treatment duration. Furthermore, the potential mechanisms by which metformin plays a role in KOA require further research to guide the development of more targeted and effective treatment strategies.

## Data Availability

The original contributions presented in the study are included in the article/Supplementary material, further inquiries can be directed to the corresponding authors.
